# High Prevalence of Enterobacterales in the Smear of Surface-Ripened Cheese with Contribution to Organoleptic Properties

**DOI:** 10.3390/foods11030361

**Published:** 2022-01-26

**Authors:** Jasmine S. Ritschard, Hanne Van Loon, Lea Amato, Leo Meile, Markus Schuppler

**Affiliations:** 1 Laboratory of Food Microbiology, Institute of Food, Nutrition and Health, ETH Zurich, Schmelzbergstrasse 7, 8092 Zurich, Switzerland; jasmine.ritschard@outlook.com (J.S.R.); hanne.vanloon@bluewin.ch (H.V.L.); 2 Laboratory of Food Biotechnology, Institute of Food, Nutrition and Health, ETH Zurich, Schmelzbergstrasse 7, 8092 Zurich, Switzerland; amatolea@yahoo.com (L.A.); leo.meile@hest.ethz.ch (L.M.)

**Keywords:** surface-ripened cheese, surface smear, red-smear, surface microbiota, Gram-negative bacteria, Enterobacterales

## Abstract

The smear of surface-ripened cheese harbors complex microbiota mainly composed of typical Gram-positive aerobic bacteria and yeast. Gram-negative bacteria are usually classified as un-wanted contaminants. In order to investigate the abundance and impact of Gram-negative bacte-ria naturally occurring in the smear of surface-ripened cheese, we performed a culture-based analysis of smear samples from 15 semi-hard surface-ripened cheese varieties. The quantity, di-versity and species distribution of Proteobacteria in the surface smear of the analyzed cheese vari-eties were unexpectedly high, and comprised a total of 22 different species. *Proteus* and *Morganella* predominated most of the analyzed cheese varieties, while *Enterobacter*, *Citrobacter*, *Hafnia* and *Serratia* were also found frequently. Further physiological characterization of *Proteus* isolates re-vealed strong proteolytic activity, and the analysis of volatiles in the smear cheese surface head-space suggested that Enterobacterales produce volatile organic flavor compounds that contribute to the organoleptic properties of surface-ripened cheese. Autochthonous members of Enterobac-terales were found in 12 of the 15 smear samples from surface-ripened cheeses, suggesting that they are part of the typical house microbiota that shape the organoleptic properties of the cheese rather than represent unwanted contaminants. However, further investigation on safety issues of the individual species should be performed in order to manage the health risk for consumers.

## 1. Introduction

The production of cheese has a long tradition in Europe that has given rise to a majority of commercially important cheese varieties [1]. About one third of the yearly cheese production of Switzerland comprises semi-hard cheeses, while the majority are surface-ripened varieties with washed rind such as Appenzeller or Raclette cheese [2]. 

The development of a complex microbial biocoenosis in the surface smear during the ripening process is characteristic of these semi-hard surface-ripened cheese varieties. This surface smear microbiota is not only responsible for the red–orange coloration of the cheese surface, it also greatly determines the organoleptic properties of this type of cheese—including its intense sulfuric smell [3]. When the equilibrium of surface smear microbiota is unbalanced, undesirable contaminants such as spoilage microorganisms or pathogens may grow on the cheese surface, which may cause rind defects or pose a potential threat to consumers’ health [4]. 

The surface smear develops naturally on cheese surface that is regularly washed with brine and ripened at suitable conditions such as 13–15 °C and high relative humidity (>95%). At the end of the ripening process, the surface smear microbiota consists mainly of salt-tolerant yeast and aerobic Gram-positive bacteria [3,5]. While bacterial count in the surface smear can exceed 10^9^ CFU cm^−2^, yeast cells are less abundant with counts of 10^5^–10^7^ CFU cm^−2^ [3,6,7]. Typical surface smear bacteria like *Arthrobacter*, *Brevibacterium*, *Corynebacterium* and *Microbacterium* may account for 50–90% of the total smear bacteria, whereas for the coagulase-negative *Staphylococcus* species, it is reported that they represent only about 1–5% on the surface of mature Tilsit-type cheese [5]. *Micrococcus* and halo-tolerant bacteria known from marine environments, such as *Halomonas* or *Marinilactibacillus*, occur usually in lower numbers in the smear of surface-ripened cheese [8,9]. However, the cheese surface smear also provides a convenient habitat for enterococci, enterobacteria and molds that are usually not expected to belong to the characteristic surface smear microbiota [10,11,12,13]. In particular, Gram-negative bacteria are frequently detected on the cheese surface as well as in the cheese matrix, but in differing abundances [14,15,16,17,18,19,20,21]. Sometimes the presence of Proteobacteria on surface-ripened cheese can result in deterioration of quality, such as purple rind defects caused by indigo- and indirubin-producing strains of *Proteus* and *Psychrobacter* [22]. In general, the outermost edible part of the cheese and the surface seem to offer more favorable conditions for the survival of Gram-negative bacteria and, in addition, are more readily accessible to environmental microorganisms [23]. Thus, the typical ‘house microbiota’ assembled by adventitious microorganisms on production equipment, such as wooden boards and shelves, milk vats, production surfaces or cheese brines, may serve as a source for Gram-negative bacteria on cheese [7,24,25]. Furthermore, raw milk cheeses might contain Gram-negative bacteria ab initio inside and upon the cheese matrix due to prior colonization of the milk during processing, whereas Gram-negative bacteria in cheese from pasteurized milk are rather derived from secondary contaminations during cheese manufacture [26,27]. 

In the literature, the abundance and function of Gram-negative bacteria in the surface smear of cheese are usually documented for individual cheese varieties [28]. Therefore, this study is intended to provide a broad overview on the topic by analyzing the diversity, abundance and function of Gram-negative bacteria in the surface smear of several varieties of semi-hard surface-ripened cheeses produced from raw and pasteurized milk. For this purpose, we monitored the proportional distribution of Gram-negative species isolated from the cheese surface smear by 16S rRNA gene amplicon sequencing. Furthermore, representative bacterial strains were tested for their physiological characteristics, such as proteolytic and lipolytic activities, in regard to cheese flavor compound production. Volatile cheese flavor compounds of the surface smear were analyzed by quantitative instrumental gas chromatography analysis in order to link the potential contribution of Gram-negative bacteria in the surface smear to flavor generation, and to discuss their role in the area of conflict between beneficial contribution to organoleptic properties and food safety aspects.

## 2. Materials and Methods

### 2.1. Cheese Samples

In total, 15 samples (V1–V15) of Swiss semi-hard surface-ripened cheeses were obtained from retail trade in Switzerland and subjected to microbial analysis of the cheese surface smear composition (Table 1). Cheese samples were acquired either as vacuum film-prepacked from self-service shelves or wrapped in cheese wrapper from the cheese counter. As indicated in Table 1, the cheese varieties included in this study were produced by different production systems and could be assigned thus: Mountain-like, Trappist-like, Raclette-like and Tilsit-like cheese. All the cheese varieties were produced from cow’s milk, either raw, after thermization (light heat treatment step, usually between 57–68 °C for 15 s) or pasteurization (72–75 °C, 30 s), and varied in fat content in dry matter from 25–55%.

### 2.2. Sampling Procedure and Microbial Analysis by Culturing

From all investigated cheeses, a rectangle of 8 cm^2^ in size and 2–3 mm thickness (corresponding to 2.3 ± 0.2 g weight) was collected from the cheese surface and further processed as described in a previous study [13]. Colony-forming units (CFU) were determined by surface plating of serial dilutions of the homogenized cheese surface smear on Tryptic Glucose Yeast Agar (TGYA; Biolife, Milano, Italy), supplemented with 1% (*w*/*v*) peptone from casein (Merck, Darmstadt, Germany), Violet Red Bile Glucose Agar (VRBG; Biolife, Milano, Italy), Endo Agar (Endo; Biolife, Milano, Italy) and Plate Count Skim Milk Agar (PCSMA; Merck, Darmstadt, Germany), supplemented with 0.0005% (*w*/*v*) crystal violet (Roth, Karlsruhe, Germany) and 0.0005% (*w*/*v*) vancomycin hydrochloride (Roth, Karlsruhe, Germany) for inhibition of Gram-positive microbiota (PCAI) as described by Delbès et al., 2007 [10]. The incubation properties were as follows: total mesophilic aerobic bacterial counts on TGYA (3 days 30 °C aerobic incubation, followed by 7 days 22 °C room temperature incubation under daylight), Gram-negative colony counts on PCAI (2 days 30 °C aerobic for PCAI or anaerobic incubation for PCAI_an_), *Enterobacteriaceae* colony counts on VRBG Agar (1 day 37 °C aerobic incubation) and coliform bacteria on Endo Agar (1 day 37 °C aerobic incubation) for isolation purposes. Colony counts on TGYA, PCAI and VRBG were determined as the weighted average.

### 2.3. Isolation of Gram-Negative Bacteria and Genotypic Identification by Partial 16S rRNA and gyrB Sequencing

Three representative colonies from each colony type identified on VRBG, PCAI, PCAI_an_ or Endo plates were picked and purified by the streak plate method on the same culture media. Two additional subcultures were performed on half-concentrated Brain Heart Infusion Agar (BHI½) containing 1.85% (*w*/*v*) BHI broth (Biolife, Milano, Italy), 0.25% (*w*/*v*) sodium chloride (Merck, Darmstadt, Germany) and 1.5% (*w*/*v*) agar (Roth, Karlsruhe, Germany). 

For identification of bacterial isolates and comparative analysis by partial 16S rDNA or *gyrB* sequencing, DNA extraction was performed from 2 mL of cells grown overnight at 37 °C in BHI½ containing 0.25% (*w*/*v*) sodium chloride. Initially, cells were washed twice in 1 mL phosphate-buffered saline (PBS) pH 7.4, containing 10 mM potassium chloride (Merck, Darmstadt, Germany), 137 mM sodium chloride (Merck, Darmstadt, Germany), 10 mM disodium hydrogen phosphate (Roth, Karlsruhe, Germany) and 2 mM potassium dihydrogen phosphate (Fluka Chemie GmbH, Buchs, Switzerland) [29]. DNA was extracted by the use of the GenElute^TM^ Bacterial Genomic DNA Kit (Sigma-Aldrich Chemie GmbH, Steinheim, Germany) according to the manufacturer’s instructions, with the exception of doubling heated incubation times and DNA elution performed with 50 µL preheated (55 °C) Milli-Q Water (Millipore AG, Zug, Switzerland). DNA concentration was determined using a ND-1000 Spectrophotometer (NanoDrop^®^ Technologies, Wilmington, USA). 

Amplification of 16S rRNA genes was performed using universal broad-range primers Forward (5′-AGA GTT TGA TCM TGG CTC AG-3′; M = A/C) and Reverse (5′-TAC CAG GGT ATC TAA TCC TGT T-3′) corresponding to positions EC8-27 and EC781-802 of the *Escherichia coli* 16S rRNA gene according to Brosius et al., 1978 [30]. Amplification of *gyrB* genes from bacterial isolates resulting in Polymerase Chain Reaction (PCR) amplicons of 1260 bp length was performed using universal primers UP1 and UP2r, as described by Yin et al., 2008 [31]. A 20 µL PCR mixture containing either 1 µL each of forward primer (10 pmol µL^−1^) and reverse primer (10 pmol µL^−1^) for 16S rRNA gene amplification, respectively 2 µL each of forward primer UP2r (10 pmol µL^−1^) and reverse primer UP1 (10 pmol µL^−1^) for *gyrB* gene amplification, 10 µL PCR Master Mix (2×) (Thermo Fisher Scientific Inc., Waltham, USA), 7 µL, respectively 5 µL, nuclease free water (Thermo Fisher Scientific Inc., Waltham, USA) and 1 µL DNA (1.5–7 ng/µL). For colony PCR, the DNA was substituted with a tiny amount of cells grown on solid media, which were transferred by a tooth pick to the PCR mixture and complemented by 1 µL of nuclease-free water. The thermocycler (Primus 25 advanced; Clemens GmbH, Waldbüttelbrunn, Germany) was programmed to perform an initial denaturation step at 95 °C for 10 min, followed by 25 cycles, each of 1 min at 95 °C, 1 min at 55 °C and 2 min at 72 °C, and a final extension at 72 °C for 10 min for 16S rRNA gene amplification, whereas PCR cycling parameters and cycle amounts for *gyrB* gene amplification were applied as proposed to be optimal by Yin et al., 2008 [32]. PCR amplicons were purified prior to sequencing through the use of a PCR clean-up kit (Sigma-Aldrich, Steinheim, Germany) in accordance with the manufacturer’s instructions. Amplicon sequencing with sequencing primer RTU3 (5′-GWA TTA CCG CGG CKG CTG-3′; W = A/T; K = G/T) (5 pmol µL^−1^) corresponding to position EC519-536, respectively with sequencing primer UP1 (10 pmol µL^−1^), was performed by GATC Biotech AG (Konstanz, Germany) [30]. 

Electropherograms of the resulting 16S rRNA or *gyrB* DNA sequences were carefully analyzed and manually edited when necessary. The sequences were compared to GenBank database sequences using the Basic Local Alignment Search Tool (BLAST) algorithm provided by the National Center for Biotechnology Information (NCBI; Bethesda, MD, USA) and SepsiTest^TM^ BLAST online tool (Molzym GmbH & Co. KG, Bremen, Germany) [32]. Best BLAST hits of database sequence comparison were recorded. Percentage values of ≥99% identity of the query sequence to the closest relative database sequence were considered as species identification, whereas percentage values between <99% and ≥97% were considered as identification on a genus level. If the two different BLAST approaches from a single sequence resulted in different species assignments, or if the BLAST results from *gyrB* and 16S rDNA sequences revealed different species assignments, the valid genus designation was recorded.

### 2.4. Determination of Proteolytic and Lipolytic Activity of Gram-Negative Isolates

Selected Gram-negative bacteria were analyzed for their proteolytic and lipolytic properties by testing hydrolysis of proteins and fats embedded in solid media. Bacteria were grown overnight in 5 mL BHI½ broth at 37 °C under aerobic conditions to a cell density of about 10^8^ cells mL^−1^, as verified by OD measurement on a spectrophotometer Libra S22 (Biochrom Ltd., Cambridge, England). For testing the proteolytic activity of isolates, 5 µL of overnight culture was spotted in triplicates on Caso Bouillon Skim Milk Agar (CBSMA) containing 4% (*w*/*v*) Caso Bouillon Broth (Roth, Karlsruhe, Germany), 5% (*w*/*v*) skim milk powder (Oxoid LTD., Basingstroke, Hampshire, England) and 1.5% (*w*/*v*) agar (Roth, Karlsruhe, Germany). Lipolytic activity was determined using 5 µL of an overnight culture spotted in triplicates on Tributyrin Agar (TBA) containing 0.5% (*w*/*v*) peptone from casein (Merck, Darmstadt, Germany), 0.3% (*w*/*v*) yeast extract (Merck, Darmstadt, Germany), 0.4% (*w*/*v*) tween 80 (Merck, Darmstadt, Germany), 1.5% (*w*/*v*) agar (Roth, Karlsruhe, Germany) and 1% (*w*/*v*) glycerintributyrate (Merck, Darmstadt, Germany). Incubation of each strain was performed under aerobic and anaerobic conditions at 4 °C, 22°C (room temperature) and 30 °C for 12 days. Proteolytic and lipolytic bacterial activity was recorded as an agar clearing zone (halo) surrounding the bacterial growth after 2, 5 and 12 days of incubation. The extent of the proteolytic or lipolytic active zone was quantified by measuring the diameter of the halo in mm and subtracting the diameter in mm of the colony formed by the tested strain. The average for three tested colonies of the same bacterial strain and the according standard deviation were calculated. Clearing zones or overgrowing colonies with a diameter of more than 40 mm were recorded as a proteolytic or lipolytic activity of >40 mm. 

### 2.5. Determination of Extended Spectrum β–Lactamase (ESBL)-Mediated Resistance of Enterobacterales Isolated from the Cheese Surface Smear

Using the disk diffusion method, antimicrobial susceptibility was tested for 27 of the previously isolated surface smear bacteria of the genera *Proteus*, *Morganella*, *Enterobacter*, *Citrobacter*, *Hafnia* and *Serratia*. Cells grown on BHI½ agar containing 0.25% (*w*/*v*) sodium chloride were diluted in 0.85% (*w*/*v*) sodium chloride solution (Biomérieux, Geneva, Switzerland) until a McFarland standard of 0.5 was reached (Vitek Densicheck; Biomérieux, Geneva, Switzerland). For confluent growth, cells were distributed equally on Mueller–Hinton Agar (Biomérieux, Geneva, Switzerland) through the use of a cotton swab (Huberlab, Aesch, Switzerland) soaked in diluted bacterial suspension. The antibiotics applied were cefoxitin (30 µg), ceftazidime (10 µg), amoxicillin with clavulanic acid (20 µg with 10 µg), cefotaxime (30 µg), cefpodoxime (10 µg), ceftriaxone (30 µg), piperacillin with tazobactam (30 µg with 6 µg) and cefepime (30 µg) (all BD BBL^TM^ Sensi-Disc^TM^; Becton Dickinson AG, Allschwil, Switzerland). Zones of antibiotic resistances were evaluated 24 h after incubation at 30 °C in accordance with current European Committee on Antimicrobial Susceptibility Testing (EUCAST) guidelines [33].

### 2.6. Volatile Organic Flavor Compound Analysis of Cheese Surface Smear

A cheese portion of Tilsit-like variety V2, obtained directly from the manufacturer at the end of the ripening period of 70–110 days, was analyzed for volatile organic flavor compounds (VOC) released from the cheese surface smear by the Institute for Food Sciences (Agroscope, Berne, Switzerland). For this purpose, volatiles deriving from the cheese surface smear were identified by headspace solid-phase microextraction coupled with gas chromatography mass spectrometry (HS-SPME-GC-MS) or gas chromatography pulsed flame-photometric detection (HS-SPME-GC/PFPD). Individual chemical compounds detected in significant amounts in the VOC profiles were compared to volatile flavor compound patterns in bibliographic data to identify chemical compounds that presumably originate from the metabolic activity of members of Enterobacterales.

## 3. Results

### 3.1. Abundance of Total Mesophilic Aerobic Bacteria, Gram-Negative Bacteria and Enterobacterales in Cheese Surface Smear

**Figure 1:**
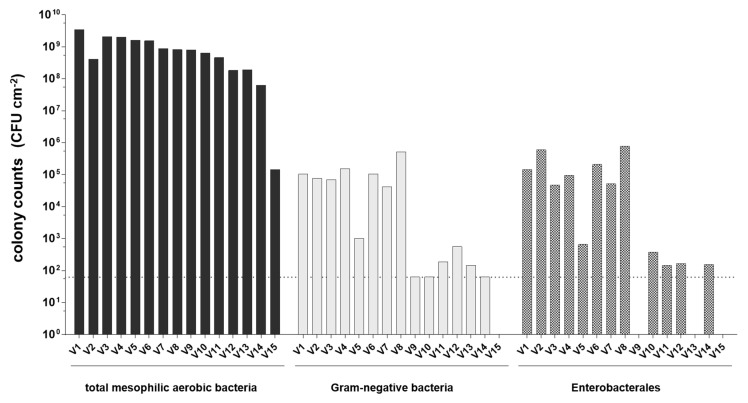
Weighted average in CFU cm^−2^ for total mesophilic aerobic bacteria, Gram-negative bacteria and Enterobacterales in the surface smear of semi-hard surface-ripened cheese varieties (V1 to V15). The detection limit is indicated by the horizontal dotted line. Lack of bars indicates samples with numbers below the detection limit.

The weighted average colony counts in CFU cm^−2^ determined by culturing of the surface smear microbiota of 15 different semi-hard surface-ripened cheese varieties are illustrated in Figure 1. Counts for total mesophilic aerobic bacteria were in the range of 6.4 × 10^7^ to 3.5 × 10^9^ CFU cm^−2^ with the exception of one sample (V15) revealing a much lower abundance of mesophilic aerobic bacteria and a lack of Gram-negative bacteria. All other smear samples contained Gram-negative bacteria in a broad range from the detection limit of 6.3 × 10^1^ CFU cm^−2^ up to 5.1 × 10^5^ CFU cm^−2^. With the exception of surface smear samples V9 and V13, all samples that contained Gram-negative bacteria harbored members of Enterobacterales in the range of 1.6 × 10^2^ CFU cm^−2^ to 7.8 × 10^5^ CFU cm^−2^. Thus, in contrast to counts for total mesophilic aerobic bacteria, the abundance of Enterobacterales and Gram-negative bacteria varied strongly in the samples of different cheese varieties.

### 3.2. Genotypic Identification of Gram-Negative Isolates by Partial Sequencing of 16S rRNA and gyrB Genes

A total of 85 Gram-negative isolates from the surface smear of the 15 surface-ripened cheese varieties were further processed for molecular identification based on partial 16S rDNA and *gyrB* sequencing (Table 2). Overall, the results revealed a high diversity comprising 22 different species that belonged to 14 different genera of Proteobacteria. Most isolates could be assigned to the γ-Proteobacteria class and 84.7% of all isolates represented Enterobacterales, whereas *Xanthomonadaceae* were represented by single isolates. Representatives of β-Proteobacteria not belonging to the order Enterobacterales comprised mainly *Alcaligenaceae* with 9.4% of total isolates and a single isolate of *Uruburuella suis* belonging to *Neisseriaceae*. Further single isolates represented members of *Caulobacteraceae* and *Phyllobacteriaceae*, which belong to α-Proteobacteria. Eight of the fifteen cheese varieties exclusively harbored members of Enterobacterales, whereas four cheese varieties additionally featured *Alcaligenaceae* or *Neisseriaceae*. The most abundant Gram-negative genera have been *Proteus* and *Morganella* with 37.7% and 23.5%, respectively, represented by the species *Proteus vulgaris*, *Proteus hauseri* and *Morganella morganii*. *Proteus* spp. were isolated from 11 of the 15 cheese varieties investigated in this study, and *Morganella* spp. were detected in 9 of the 15 cheese varieties (Table 2). Further frequent isolates corresponded to *Citrobacter* sp. (9.4%), *Enterobacter* sp. (5.9%), and *Serratia* sp. (3.5%). *Providencia* sp., *Providencia heimbachae*, *Providencia rettgeri* and *Serratia proteamaculans* were represented by single isolates only. *Escherichia coli*, as a typical fecal indicator, was not present. The family *Alcaligenaceae* was represented by *Pusillimonas* sp. (4.7%), *Advenella* sp. (2.4%) and a single isolate of *Kerstersia gyiorum.*

Only two cheese varieties (V9 and V13) that harbored Gram-negative bacteria did not reveal a predomination of Enterobacterales. Cheese variety V9 harbored *Stenotrophomonas maltophilia* as the sole representative of Proteobacteria, whereas the surface microbiota of cheese variety V13 revealed a wide variety of different Proteobacteria, including *Advenella* sp., *Advenella kashmirensis*, *Brevundimonas diminuta*, *Defluvibacter*, *Pusillimonas* and *Stenotrophomonas*. In general, the numbers for different genera of Proteobacteria determined in the surface smear samples varied between 10^1^ CFU cm^−2^ for the genera *Brevundimonas* and *Defluvibacter* and 10^4^ CFU cm^−2^ for *Advenella*, *Citrobacter*, *Morganella*, *Proteus*, *Providencia* and *Pusillimonas*. *Enterobacter*, *Hafnia*, *Kerstersia*, *Serratia*, *Stenotrophomonas* and *Uruburuella* were represented with 10^2^ to 10^3^ CFU cm^−2^.

### 3.3. Proteolytic and Lipolytic Activities of Isolated Proteobacteria

Many representatives of Enterobacterales are known to contribute to the formation of key aroma compounds in cheese by their enzymatic activity [34]. Consequently, 14 selected surface smear isolates from cheese varieties V2, V3 and V5 were tested for their proteolytic and lipolytic potential by applying a spot-on-the-lawn test. Only *Proteus* isolates revealed proteolytic activity in situ on Caso Bouillon Skim Milk Agar (Table 3) with isolates V2.4, V2.8 and V5.3 showing highest proteolytic activity, as the whole CBSMA Agar plate was cleared at the latest after 5 days of incubation. Three *Proteus* isolates (V2.5, V2.8 and V5.3) revealed proteolytic activity also under anaerobic conditions, but only after 12 d of incubation (data not shown).

Lipolytic activity of isolates was tested on Tributyrin Agar (TBA). In general, lipolytic activity was much less frequently observed than proteolytic activity. *Halomonas* and *Proteus* isolates from cheese sample V2 revealed lipolytic activity after 12 days’ aerobic incubation at room temperature and 30 °C, whereas a *Providencia heimbachae* isolate from cheese sample V5 showed lipolytic activity in vitro after 12 days at 30 °C. No lipolytic activity could be observed under anaerobic growth conditions.

### 3.4. Volatile Carbonic Flavor Compounds Produced by Gram-Negative Isolates of the Cheese Surface Smear

To investigate the potential contribution of Proteobacteria to the typical flavor of semi-hard surface-ripened cheese, a headspace atmosphere analysis was performed on cheese variety V2, which was known to harbor different Proteobacteria such as *Advenella*, *Morganella*, *Proteus*, *Providencia* and *Pusillimonas*. Application of HS-SPME-GC-MS and HS-SPME-GC/PFPD revealed a variety of different volatile carbonic flavor compounds such as alcohols, aldehydes, carboxylic acids, ketones and sulfur compounds (data not shown). The majority of aroma compounds detected by the analysis were sulfur compounds (dimethyl sulfide, dimethyl disulfide, dimethyl trisulfide, hydrogen sulfide, methanethiol and methylthioacetate), followed by carboxylic acids (acetic acid, butyric acid, formic acid and 3-methylbutyric acid) and ketones (acetoin, 2-butanone, 2-nonanone and 1-phenylethanone). Alcohol compounds determined in the headspace comprised 3-methylbutanol and phenylethanol. Interestingly, many of the volatile organic compounds detected in significant amounts by headspace atmosphere analysis of cheese variety V2 are known to be produced by representatives of Enterobacterales [14,16,19,35,36,37,38,39,40]. 

### 3.5. Extended Spectrum β–Lactamase (ESBL)-Mediated Resistances of Enterobacterales Isolated from the Cheese Surface Smear

To investigate potential health risk factors of individual smear bacteria, selected isolates that come into consideration as ESBL producers were tested for their antibiotic susceptibility and the ability to produce extended spectrum β–lactamase. However, none of the *Morganella*, *Enterobacter*, *Citrobacter*, *Proteus*, *Hafnia* and *Serratia* isolates featured ESBL production, whereas all the *Morganella* isolates as well as one isolate each of the genera *Enterobacter*, *Citrobacter* and *Hafnia* expressed AmpC β-lactamase, which is intrinsically present in these species. 

## 4. Discussion 

The present study revealed an unexpectedly high abundance and diversity of Proteobacteria species on the surface of different semi-hard surface-ripened cheese varieties at the end of the ripening stage. The composition of Gram-negative bacterial population of the surface microbiota varied considerably between different cheese varieties. High counts of up to 7.8 × 10^5^ CFU cm^−2^ Gram-negative bacteria were determined for the majority of cheese varieties analyzed in the present study. These values were comparable to counts of approx. 10^5^ CFU cm^−2^ Gram-negative bacteria determined for Tilsit-type cheeses as reported in the literature for [5,13]. Differences in the quantity of Gram-negative bacteria might be influenced by differing production technologies, seasonal variation or the milk type applied for cheese production. With up to 10^9^ CFU cm^−2^, the quantity of total aerobic mesophilic bacteria in cheese smear was very similar for all cheese varieties analyzed in this study, and corresponded well to values reported for various surface-ripened cheese varieties in the literature [3,5,6,8].

At least 22 different species of Proteobacteria were isolated, belonging to 14 different genera. With 84.7% of the 85 identified isolates, Enterobacterales represented the most frequent Gram-negative bacteria. About half of the analyzed cheese varieties featured exclusively members of Enterobacterales, whereas only two varieties lacked its representatives. The observed predomination of Enterobacterales as major representatives of Gram-negative bacteria isolated from cheese surface microbiota is consistent with data from previous studies on surface-ripened cheese [12,41]. In this study, Enterobacterales were mainly represented by isolates of the genera *Proteus* and *Morganella*, whereas *Citrobacter*, *Enterobacter*, *Serratia*, *Providencia* and *Hafnia* were isolated to a much lesser extent. Besides Enterobacterales, Gram-negative bacteria regularly detected on the surface of surface-ripened cheeses belonged mainly to Proteobacteria families such as *Alcaligenaceae*, *Caulobacteraceae*, *Halomonadaceae*, *Moraxellaceae*, *Oceanospirillaceae*, *Pseudoalteromonadaceae*, *Pseudomonadaceae*, *Vibrionaceae* and *Xanthomonadaceae* [12,13,15,20,24,42,43,44]. On Livarot, a French surface-ripened soft cheese, Gram-negative bacteria accounted for 32% of the overall bacteria isolated from the cheese surface [15]. A recent study on the temporal differences in the microbial composition of Époisses cheese rinds during ripening and storage also revealed a dominance of Gram-negative species. At the end of ripening, about 70% of all 16S rRNA gene sequences were derived from Gram-negative bacteria, with the most abundant genera being *Psychrobacter*, *Halomonas*, *Mesonia* and *Vibrio* [28].

Proteobacteria other than Enterobacterales isolated from the surface of cheese varieties investigated in this study were mainly represented by single isolates, and comprised members of *Alcaligenaceae* (*Advenella*, *Kerstersia* and *Pusillimonas*) as well as *Caulobacteraceae* (*Brevundimonas*), *Phyllobacteriaceae* (*Defluvibacter*), *Neisseriaceae* (*Uruburuella*) and *Xanthomonadaceae* (*Stenotrophomonas*). *Brevundimonas diminuta* and *Stenotrophomonas* sp. were already reported to be part of the surface smear microbiota of different surface-ripened French cheeses [12]. *Advenella kashmirensis* as well as *Pusillimonas* sp. have been described for Austrian mountain cheese [45]. To our best knowledge, for the genera *Defluvibacter*, *Kerstersia* and *Uruburuella* this is the first description of an association with cheese. *Defluvibacter* sp. represents typical environmental bacteria isolated from aqueous habitats such as the activated sludge of waste water plants [46]. In contrast, *Kerstersia gyiorum* has been isolated from various human clinical samples such as wounds, feces or chronic infection sites [47]. Although the definite pathogenic potential has not been reported for this species, its contribution to chronic infections is discussed [47]. Likewise, *Uruburuella suis*, isolated first from clinical specimens of animals, seems to be related to respiratory diseases in pigs [48]. 

Our study revealed no correlation between the abundance of Gram-negative bacteria or species diversity and the type of milk applied for cheese production, whether raw, thermized or pasteurized. This is consistent with the finding that on an average, similar percentages of Proteobacteria were determined in raw milk and pasteurized milk cheese using high-throughput sequencing analysis of 62 different cheese samples [20]. Furthermore, the type of production, whether artisanal manufacturing, traditional or mass production, did not influence the quantity and diversity of Proteobacteria. It is reported that members of Proteobacteria populate functional habitats such as milk handling surfaces or cheese maturation surfaces in artisanal cheese manufacturing plants [24]. In particular, coliform bacteria and *Pseudomonadaceae* are known to be part of biofilms on wooden vats and shelves from which inoculation might take place during cheese production or ripening [25]. Halotolerant Proteobacteria such as *Psychrobacter*, *Pseudoalteromonas* and *Vibrio* are supposed to establish a ‘house microbiota’, as they are similarly abundant in aging rooms and cheese surfaces [24]. While cheeses produced from pasteurized milk might exclusively be colonized by a secondary contamination with Proteobacteria, those produced from raw or thermized milk might contain indigenous Proteobacteria originating from the milk. Raw milk can be populated by significant proportions of mainly psychrotrophic Proteobacteria (*Enterobacteriaceae*, *Pseudomonas*, *Acinetobacter*) that might flourish during cold storage, and even dominate dairy tank milk [27,42]. As Gram-negative bacteria are known to be adherent to stainless steel and therefore exhibit a high biofilm-forming potential, they are able to colonize dairy pipelines leading to potential recontamination of pasteurized milk [26]. However, for San Simón cheese it was demonstrated that counts for *Enterobacteriaceae* in the surface smear constantly decreased during a 6-week ripening period, which was related to the influence of the combined effects of physico-chemical parameters [19].

It is assumed that members of Enterobacterales contribute to typical cheese flavor development by the lipolytic breakdown of milk fat and the generation of semi-volatile fatty acids and the according ethyl esters, while peptidase and deaminase activity on proteins contributes to the production of volatile aroma compounds [35,40]. In this study, lipolytic activity was observed for isolates of *Halomonas* sp., *Proteus* sp., and *Providencia heimbachae*, which seems to be the first description of lipolytic activity for *Halomonas* and *Providencia*. According to the literature, *Citrobacter* spp. and *Enterobacter* spp. may also contribute to aroma compound production in cheese although their proteolytic and/or lipolytic activity in vitro seems to be less pronounced [49].

*Proteus* spp., in particular *Proteus vulgaris* strains, were frequently isolated from the surface of surface-ripened cheeses, and their potential role in cheese ripening has already been intensively discussed [12,13,14,15,36,40,50]. *Proteus vulgaris* was able to successfully colonize and dominate the surface of pilot-scale cheese while significantly contributing to its organoleptic properties through the production of aldehydes [14]. Therefore, the presence of *Proteus vulgaris* might be desirable for the development of typical aroma compounds [50]. In agreement with the high caseinolytic activity reported for crude culture supernatants of *Proteus vulgaris*, all *Proteus* strains investigated in this study featured strong proteolytic activity [40]. In this study, Enterobacterales isolates other than *Proteus* spp. showed no casein degradation, although this was described for cheese smear isolates affiliated to the genera *Enterobacter* or *Citrobacter* [16,34,49]. Accordingly, this enzymatic activity seems to be a strain-dependent feature [34]. In experimental cheese produced from milk artificially inoculated with *Enterobacteriaceae*, the degradation of major casein fractions could be assigned to their specific proteolytic activity [34].

Enzyme-driven catabolic activities such as proteolysis and the subsequent catabolism of amino acids or lipolysis, besides lactose and citrate metabolism implying glycolysis, impact to a great extent the organoleptic properties of cheese [51,52]. In the literature, various Proteobacteria species are described to produce a wide variety of volatile compounds in large quantities when growing in milk, cheese model medium or cheese surface smear, thereby impacting cheese flavor during ripening [14,16,36,37,40]. A variety of the aroma compounds typical for cheese flavor were detected in significant amounts in the volatile compound pattern of the headspace atmosphere of cheese variety V2 analyzed in this study. Mainly volatile compounds such as carboxylic acids, ketones and sulfur compounds could be related to Enterobacterales isolates after comparing the results to bibliographic data. As *Morganella* and *Proteus* spp. predominated the Gram-negative fraction of isolates from the surface smear of cheese variety V2, we concluded that they may contribute to the respective volatile aroma compounds detected in the headspace atmosphere of cheese variety V2. In particular, *Proteus vulgaris* is known to produce a wide variety of volatile compounds with low perception threshold in large amounts (dimethyl disulfide, 3-methylbutanol) in cheese model medium, and has a prevalence on aroma compound production during the ripening of a model cheese [14,40]. A *Proteus vulgaris* isolate from the surface of surface-ripened French cheese was able to produce inter alia dimethyl-trisulfide, dimethyl-sulfide, 2-butanone, 1-phenylethanone and phenylethanol in liquid medium [36]. These volatile compounds were also detected in the headspace of cheese variety V2, providing further evidence that *Proteus* sp. may contribute to the formation of the volatile sulfur compounds that represent garlic odor notes known to be increasingly produced at the end of the ripening process [40]. This assumption is supported by a study which indicates that using human senses (odor and taste), it is possible to predict high levels of *Enterobacteriaceae* in soft cheeses made from raw milk [53]. 

As *Proteus vulgaris* and *Hafnia alvei* produce a wide variety and large quantity of volatile compounds, non-pathogenic and non-biogenic amine-producing strains thereof were proposed as industrially applicable microorganisms for the production of natural cheese flavor compounds [49]. *Hafnia alvei* inoculated in pilot-scale cheese was able to establish successfully at the beginning of the ripening process in the cheese community and clearly intensified the production of volatile compounds, mainly by production of volatile sulfur compounds [37]. Hence, non-pathogenic strains of *Hafnia alvei* are commercially available for the purpose of conferring a flavor comparable to raw milk cheese to that produced from pasteurized milk [37]. Accordingly, as one of few Enterobacterales, *Hafnia alvei* was included in an authoritative list of microbial food cultures with practical use by the International Dairy Federation (IDF) [54]. However, the potential for production of biogenic amines in vitro or the presence of antibiotic resistance genes might question the use of such bacteria in cheese production [12,16,17]. 

Concerning the presence of Proteobacteria in food products such as cheese, the positive as well as negative aspects considering the risks and benefits of Gram-negative bacteria in cheese surface smear are up for debate. Many traditional cheeses harbor Proteobacteria, which are considered to belong to natural autochthonous microbiota and are thought to have a beneficial impact on cheese sensory characteristics [14,15,16,17,41]. However, a previous study on the development of a defective smear after prepackaging of surface-ripened cheese in plastic foil revealed that the presence of certain members of Proteobacteria may take a turn for the worse. The vacuum foil prepackaging of surface-ripened cheese can result in a shift in the microbial composition of the smear microbiota towards Proteobacteria, which would then contribute to the development of negative organoleptic properties in the surface smear of the cheese [55]. Furthermore, certain members of Enterobacterales found in cheese are regarded as markers for unsatisfactory hygiene practice during food processing due to their relation to bacterial strains of fecal origin [56]. Threshold and tolerance values for the number of indicator microorganisms revealing bacteriological safety exist in the food legislation of almost all European countries. In the case of Swiss cheese, Swiss food hygiene legislation prescribes limit values for *Escherichia coli* in cheese produced from heat-treated milk but not for raw milk cheeses. However, limit values are defined for *Salmonella* spp. as food safety criteria in cheeses produced from raw milk or milk with a heat treatment below pasteurization temperature (thermized milk). Accordingly, as no *Escherichia coli* or *Salmonella* spp. were isolated from the cheese surface, the occurrence of *Citrobacter*, *Enterobacter*, *Hafnia*, *Morganella*, *Proteus*, *Providencia* and *Serratia* in cheese smear is in agreement with current regulations. 

Beside hygiene-related aspects, food safety issues challenge the occurrence of Gram-negative bacteria in cheese. According to the European Food Safety Authority (EFSA), none of the Gram-negative bacterial species identified in this study qualified for presumption of safety (QPS) status when intended as biological culture added to foods [57]. Food-borne outbreaks related to Gram-negative bacteria in cheese are rare, and cover mainly *Salmonella*, enteropathogenic or shiga toxin-producing *E*. *coli* or *Brucella* spp. [58]. Intense hygienic efforts and improved technology applied in the dairy process, such as pasteurization and proper acidification of milk during cheese production as well as the preservation of semi-hard cheese by salting, minimize the risk for transfer of food-borne pathogens. Furthermore, certain strains of Gram-negative species exhibited an antilisterial effect in vitro and in situ in previous studies [59]. Though several Proteobacteria species isolated in this study (*Brevundimonas diminuta*, *Citrobacter* sp., *Enterobacter* sp., *Hafnia alvei*, *Morganella morganii*, *Proteus vulgaris*, *Stenotrophomonas maltophilia*, *Serratia proteamaculans*) represent opportunistic pathogens (classified to biosafety level 2), they are associated with diseases that are rarely serious. 

Antibiotic resistances such as extended spectrum β-lactamase (ESBL)-mediated resistance of *Enterobacteriaceae* concern a further safety issue. Several isolates of Enterobacterales from this study have been tested for extended spectrum β-lactamase (ESBL) production, but none of the isolates exhibited such a resistance. The detected AmpC β-lactamases are intrinsically present in many species. They are chromosomally encoded and usually expressed at low levels [60,61]. Considering the literature about ESBL producing *Enterobacteriaceae* related to cheese, potential β-lactam resistance was detected mainly in *Escherichia coli* strains isolated from cheese produced in different countries [60]. As the prevalence of ESBL in Europe is high, the emergence of antibiotic resistance in food-related bacteria needs to be closely monitored [62]. Although the consumption of antibiotic-resistant *Enterobacteriaceae* may not present an immediate risk to human health, antibiotic-resistant genes might be transferred from commensal bacteria to obligate pathogenic bacteria on the cheese surface or bacteria of the human intestinal microbiota [12]. 

## 5. Conclusions

This study provides valuable insights into the occurrence of Enterobacterales in cheese surface microbiota and their presumable contribution to flavor development in cheese. An unexpectedly high diversity of Gram-negative bacteria was detected in the smears of different semi-hard surface-ripened cheese varieties. *Proteus* spp. and *Morganella* spp. were most prominent, independent of the milk type applied for cheese production or the type of manufacturing process. The quantity of Gram-negative bacteria in the surface smear differed between different cheese varieties, and could have been influenced by dairy ‘house microbiota’. *Proteus* spp. seemed to contribute in particular to the organoleptic properties of surface-ripened cheese by their strong proteolytic activity. Furthermore, a variety of volatile flavor compounds with low perception threshold was detected in the headspace atmosphere of the surface of cheese variety V2, which most likely resulted from the enzymatic activity of Enterobacterales. On the other hand, several isolated Enterobacterales species represented opportunistic pathogens. Therefore, safety issues related to these species need to be investigated in order to manage the risk to consumer health. 

In conclusion, cheese manufacturers are encouraged to produce as tasteful a cheese as possible, with an appealing appearance and of high quality, stability and safety, independent of the seasonal variability induced in basic raw material. Autochthonous-occurring Enterobacterales on the cheese surface appear to be valuable for the organoleptic properties of various cheese varieties, and must not entirely be considered as undesired contaminants. Naturally occurring surface smear microbiota comprising Enterobacterales may protect the surface against unwanted mold spoilage or pathogenic contaminants. Consequently, abandonment of Enterobacterales from the surface smear due to food safety reasons might result in cheese lacking the typical flavor that is important for the authenticity of its variety.

## Figures and Tables

**Table 1:** Specifications of semi-hard surface-ripened cheese samples analyzed in this study.

Sample	Cheese Type	Weight ^a^	Milk Type ^b^	Fat Content ^c^	Production Type
V1	Trappist-like	0.4 kg	pasteurized	45%	artisanal
V2	Tilsit-like	4 kg	thermized	45%	traditional
V3	Tilsit-like	4 kg	pasteurized	45%	traditional
V4	Tilsit-like (mild/creamy)	4 kg	pasteurized	45%	traditional
V5	Trappist-like	0.8 kg	thermized	51%	traditional
V6	Raclette-like	4 kg	thermized	25%	artisanal
V7	Raclette-like	5 kg	pasteurized	30%	artisanal
V8	Tilsit-like (mild/creamy)	3 kg	thermized	53%	artisanal
V9	Mountain cheese-like	5 kg	thermized	45%	artisanal
V10	Tilsit-like (mild/creamy)	6 kg	pasteurized	31%	traditional
V11	Tilsit-like	7 kg	raw milk	48%	traditional
V12	Tilsit-like (mild/creamy)	4 kg	pasteurized	55%	industrial
V13	Tilsit-like	7 kg	raw milk	45%	traditional
V14	Raclette-like	8 kg	raw milk	51–54%	traditional
V15	Tilsit-like (mild/creamy)	2 kg	pasteurized	45%	industrial

^a^ weight of a whole round cheese wheel according to cheese manufacturer; ^b^ according to cheese manufacturer; ^c^ in dry matter according to cheese manufacturer.

**Table 2:** Overview of Gram-negative bacteria identified in the smear of semi-hard surface-ripened cheese samples V1 to V15.

	Occurrence in Surface Smear of Cheese Sample															
	V1	V2	V3	V4	V5	V6	V7	V8	V9	V10	V11	V12	V13	V14	V15	
Bacterial Species																Isolation Media
																
*Advenella* sp.	−	**+**	−	−	−	−	−	−	−	−	−	−	**+**	−	−	VRBG/PCAI
*Advenella kashmirensis*	−	−	−	−	−	−	−	−	−	−	−	−	**+**	−	−	Endo
*Brevundimonas diminuta*	−	−	−	−	−	−	−	−	−	−	−	−	**+**	−	−	PCAI
*Citrobacter* sp.	**+**	−	−	**+**	−	−	**+**	−	−	−	−	−	−	−	−	VRBG/PCAI/PCAI_an_/Endo
*Defluvibacter* sp.	−	−	−	−	−	−	−	−	−	−	−	−	**+**	−	−	VRBG
*Enterobacter* spp.	**+**	−	−	**+**	−	−	**+**	−	−	−	−	−	−	−	−	VRBG/PCAI/PCAI_an_/Endo
*Hafnia alvei*	−	−	−	−	−	**+**	−	−	−	−	−	−	−	−	−	PCAI_an_
*Kerstersia gyiorum*	−	−	−	−	−	−	−	−	−	−	**+**	−	−	−	−	Endo
*Morganella* spp.	**+**	−	**+**	−	**+**	−	−	−	−	−	−	−	−	−	−	VRBG/PCAI/Endo
*Morganella morganii*	**+**	**+**	**+**	**+**	−	−	**+**	**+**	−	**+**	**+**	−	−	−	−	VRBG/PCAI/PCAI_an_/Endo
*Proteus* spp.	−	**+**	−	**+**	**+**	**+**	**+**	**+**	−	**+**	**+**	**+**	−	**+**	−	VRBG/PCAI/Endo
*Proteus hauseri*	−	**+**	**+**	−	−	−	−	−	−	−	−	−	−	−	−	VRBG/PCAI
*Proteus vulgaris*	−	**+**	**+**	−	−	**+**	−	−	−	−	−	−	−	−	−	VRBG/PCAI
*Providencia* sp.	−	**+**	−	−	−	−	−	−	−	−	−	−	−	−	−	VRBG
*Providencia heimbachae*	−	−	−	−	**+**	−	−	−	−	−	−	−	−	−	−	VRBG
*Providencia rettgeri*	−	**+**	−	−	−	−	−	−	−	−	−	−	−	−	−	VRBG
*Pusillimonas* spp.	−	**+**	−	−	−	−	−	−	−	−	−	−	**+**	**+**	−	VRBG/Endo
*Serratia* spp.	−	−	−	**+**	−	−	−	**+**	−	−	−	−	−	−	−	PCAI/PCAI_an_
*Serratia proteamaculans*	−	−	−	**+**	−	−	−	−	−	−	−	−	−	−	−	PCAI_an_
*Stenotrophomonas* sp.	−	−	−	−	−	−	−	−	−	−	−	−	**+**	−	−	PCAI
*S. maltophilia*	−	−	−	−	−	−	−	−	**+**	−	−	−	−	−	−	PCAI
*Uruburuella suis*	−	−	−	−	−	−	−	**+**	−	−	−	−	−	−	−	PCAI

Endo, Endo Agar; PCAI, Plate Count Skim Milk Agar supplemented with crystal violet and vancomycin-hydrochloride (PCAI_an_, PCAI anaerobic incubation); VRBG, Violet Red Bile Glucose Agar.

**Table 3:** Proteolytic activity of selected *Proteobacteria* isolates under different environmental conditions.

		Proteolysis Zone (mm) ^a^					
		Aerobic Conditions					
		RT			30 °C		
Isolate No.	Species	2 d	5 d	12 d	2 d	5 d	12 d
V2.4	*Proteus* sp.	29 ± 2	>40	>40	27 ± 5	>40	>40
V2.5	*Proteus* sp.	4 ± 2	3 ± 1	>40	7 ± 1	2	>40
V2.6	*Proteus* sp.	-	5 ± 1	12 ± 4	-	2 ± 1	11 ±6
V2.7	*Proteus* sp.	2 ± 1	19 ± 2	>40	3 ± 1	>40	>40
V2.8	*Proteus* sp.	38 ± 4	>40	>40	-	>40	>40
V2.9	*Proteus* sp.	-	6 ± 2	20 ± 5	2 ± 1	7 ± 2	25 ± 2
V5.3	*Proteus* sp.	>40	>40	>40	>40	>40	>40

^a^ The proteolysis zone in mm was defined as the diameter of the spot showing clearance due to proteolysis after subtraction of the diameter of the grown colony. RT, room temperature (22 °C); d, days of incubation.

## Data Availability

Data supporting the reported results can be found on the ETH Zurich Research Collection: https://doi.org/10.3929/ethz-a-010476499.
